# Parents’ experiences of participating in the Healthy School Start Plus programme – a qualitative study

**DOI:** 10.1186/s12889-023-15552-8

**Published:** 2023-04-04

**Authors:** Mahnoush Etminan Malek, Susanne Andermo, Gisela Nyberg, Liselotte Schäfer Elinder, Emma Patterson, Åsa Norman

**Affiliations:** 1 Department of Global Public Health, Karolinska Institutet, Tomtebodavägen 18A, Stockholm, 171 77 Sweden; 2 Department of Neurobiology, Care Sciences and Society, Karolinska Institutet, Stockholm, Sweden; 3 The Swedish School of Sport and Health Sciences (GIH), Lidingövägen 1, Stockholm, 114 33 Sweden; 4 Centre for Epidemiology and Community Medicine, Region Stockholm, Solnavägen 1E, Stockholm, 113 65 Sweden; 5 Section for Risk and Benefit Assessment, Swedish Food Agency, Box 622, Uppsala, 751 26 Sweden; 6 Department of Clinical Neurosciences, Karolinska Institutet, Stockholm, 171 77 Sweden; 7 Department of Psychology, Stockholm University, Stockholm, 106 91 Sweden

**Keywords:** Diet, Physical activity, Socioeconomic position, Elementary school, Parental involvement, Evaluation

## Abstract

**Background:**

The rise in overweight and obesity among children is a global problem and effective prevention interventions are urgently required. Parents play an important role in children’s lifestyle behaviours and body weight development and therefore there is a great need to investigate how to involve parents effectively in health promotion and prevention programmes. The aim of the study was to describe parents’ experiences of barriers and facilitators of participating in the Healthy School Start Plus (HSSP) intervention study.

**Methods:**

HSSP is a parental support programme, conducted in Sweden, with the aim to promote a healthy diet, physical activity and preventing obesity in 5-7-year-old children starting school. In total 20 parents from 7 schools participated in semi-structured telephone-based interviews. The data was analysed using qualitative content analysis, with a deductive approach based on the Consolidated Framework for Implementation Research (CFIR).

**Results:**

Parental experiences of barriers and facilitators informing the implementation of the HSSP intervention were identified within all five domains of the CFIR. Two additional constructs, not included in the CFIR were identified: Social factors and Cooperation. The findings of parental experiences of barriers and facilitators related to the importance of (1) adaptation of the intervention to fit the abilities of the parents with different social and cultural backgrounds; (2) the need for continuous delivery of information related to healthy behaviours; (3) the commitment and efforts of the deliverers of the intervention; (4) the need for repetition of information related to healthy behaviours given by the deliverers of the intervention; (5) encouragement and facilitation of the involvement of the family and key people around them through the intervention activities and by the deliverers of the intervention; (6) awareness of unexpected impacts and social and cultural conditions complicating the execution of the intervention and; (7) cooperation and a well-functioning interaction between parents and school staff.

**Conclusions:**

Barriers and facilitators indicated by the parents highlighted that interventions like the HSSP need to be adapted to fit the parents’ abilities, with reminders, follow-ups and delivery of relevant information. Variations in social and cultural conditions need to be taken into consideration. The commitment of the school and the interaction between the school staff and the family as well as key people around them appears to be important.

**Trial registration:**

The Healthy School Start Plus trial was retrospectively registered in the International Standard Randomised Controlled Trial Number Registry on January 4, 2018 and available online at ClinicalTrials.gov: No. NCT03390725.

**Supplementary Information:**

The online version contains supplementary material available at 10.1186/s12889-023-15552-8.

## Background

There is an urgent need for effective prevention interventions against the rising prevalence of overweight and obesity worldwide [1, 2]. Children with obesity face both physical and emotional health consequences and if the unhealthy weight is maintained into older age, it may lead to e.g., cardiovascular diseases, type-2 diabetes (T2D) and certain cancers [3–5]. The rise in BMI in children and adolescents has levelled off in several high-income countries [2], however social inequalities in overweight and obesity remain strong to the disadvantage of children from families with a low socioeconomic position (SEP) [6]. Therefore, effective health promoting and obesity preventing programmes with the aim to reach families in socially disadvantaged areas are highly needed [7–9].

To prevent overweight and obesity, there is evidence for effectiveness of interventions which target diet and physical activity and which start at an early age [10]. Further, the literature suggest that these interventions should be multi-component, involve parents and be implemented in child health care and in schools [11–16]. The possibility to reach all children above 6 years regardless of their SEP or weight status makes the school setting ideal for such universal interventions which do not seem to increase health inequalities or result in other adverse effects [10].

The importance of the involvement of parents in school-based obesity prevention interventions has been emphasised, and the need for more research in non-school settings has been highlighted [11]. Direct involvement of parents rather than just sending home health information has been shown to lead to stronger preventive effects in both school, child health care, and primary health care settings [17–21]. The reason for this is that younger children’s diet, physical activity and weight development are strongly dependent on their parents [22, 23]. Studies indicate that parents might be encouraged to influence their children’s weight-related behaviours if they themselves recognise the importance of it [22, 24]. In order to illuminate what works, in what context, why and for whom, process evaluations which include detailed contextual information are needed [25].

The aim of this study was to describe parents’ experiences of barriers and facilitators related to participating in the Healthy School Start Plus (HSSP) intervention [26] using the Consolidated Framework for Implementation Research (CFIR) [27] as guidance. An advantage of employing this widely used framework is that results become comparable to other studies using it, and that the knowledge generated can be transferred to other parental support interventions. In Sweden children up to the age of six attend the Swedish child health care which is free of charge [28].The children start pre-school class at the age of six and are regularly measured by the school nurse in the school health care, which also is free of charge. This study is part of the third cluster-randomised trial of this parental support programme performed in schools in disadvantaged areas in the Stockholm region with the overall aim to promote healthy dietary and physical activity behaviours and prevent unhealthy weight development among children. The key components of the programme are: (1) A health information brochure for parents; (2) Motivational interviewing (MI) sessions for parents; (3) Classroom activities for children and a workbook; and (4) A self-test of T2D risk for parents, the FINDRISC test [26]. Process evaluations of the earlier versions of the programme [29, 30] have found that it is important to highlight parents as role models as well as how they cooperate with each other to achieve changes in the home environment. Furthermore, it was highlighted that adapting the intervention to the abilities of the parents is important in order to enhance engagement [30].

This study will add further knowledge regarding how best to reach and involve parents to increase intervention effectiveness and the findings may be relevant for planning and scaling-up of similar school-based programmes in disadvantaged areas. These results will be relevant for the scaling up of the HSSP programme [31], as the findings will be taken into account.

## Methods

A qualitative design was used to explore the views and experiences of the parents who participated in the HSSP intervention. The interview guide was based on the CFIR [27] and results were analysed with a deductive approach. A qualitative design is suitable for studying issues in depth and for process evaluations [32]. The CFIR framework has unified constructs from 19 implementation theories, models and frameworks and presents consistent terminology and definitions across various contexts. CFIR compiles a total of 39 constructs of importance for successful implementation in five overarching domains [27]. The CFIR domain Characteristics of Individuals represents the deliverer’s perspective, i.e., the practitioner delivering the intervention. However, in the HSSP both school staff and parents can be considered to be deliverers, as the school staff deliver the intervention to the parents, who in turn, deliver it to the child. Therefore, in this study the domain Characteristics of Individuals, takes the perspective of the parent as the deliverer, and thus represents characteristics of the parents. Furthermore, as the total of constructs described in CFIR are as many as 37, only the constructs that were deductively identified are described. Table 1 shows the identified CFIR constructs and how they were applied to the data of the study in the deductive analysis in this study.

**Table 1:** Description of the application of CFIR constructs identified in the current study

CFIR *Domains* & Constructs	Description of constructs	Application of CFIR constructs in the current study focusing on the Healthy School Start Plus
***Intervention Characteristics***		
**Evidence Strength & Quality**	Stakeholders’ perceptions of the quality and validity of evidence supporting the belief that the innovation will have desired outcomes.	Parents’ confidence in the legitimacy and quality of the results of the intervention.
**Adaptability**	The degree to which an innovation can be adapted, tailored, refined, or reinvented to meet local needs.	Parents’ perceptions of the flexibility of the intervention and the level of which it can be tailored to fit specific needs of the family.
**Complexity**	Perceived difficulty of the innovation, reflected by duration, scope, radicalness, disruptiveness, centrality, and intricacy and number of steps required to implement.	Parents’ perceptions of the level of complexity of the intervention components.
**Design Quality & Packaging**	Perceived excellence in how the innovation is bundled, presented, and assembled.	Parents’ perceptions of the packaging and the presentation of the intervention, related to both the material and the deliverers.
***Outer Setting***		
**Needs & Resources of Those Served by the Organization**	The extent to which the needs of those served by the organization (e.g., patients), as well as barriers and facilitators to meet those needs, are accurately known and prioritized by the organization.	The needs and wishes expressed by parents to work on their children’s dietary and activity habits.
***Inner setting***		
**Available Resources**	The level of organizational resources dedicated for implementation and on-going operations including physical space and time.	The degree of resources dedicated from the school for the intervention.
***Characteristics of Individuals***		
**Knowledge & Beliefs about the Innovation**	Individuals’ attitudes toward and value placed on the innovation, as well as familiarity with facts, truths, and principles related to the innovation.	Parents’ values and attitudes placed on the intervention as a whole.
***Process***		
**Executing**	Carrying out or accomplishing the implementation according to plan.	Parents’ expressions about which family members who have participated in and executed the different parts of the intervention.

Description of CFIR domains and constructs from Damschroder LJ et al. [27].

### Setting and participants

The participants of this study consisted of parents in the intervention group of the HSSP trial, conducted from November 2017 to May 2018. The interviews were conducted after the end of the intervention in June 2018. The HSSP is a parental support programme with the overall aim to promote healthy physical activity and dietary behaviours, prevent unhealthy weight development in pre-school class children (5-7-year-olds), with a special focus on behaviours in the home setting. A detailed description of HSSP, which builds on Social Cognitive Theory can be found in the study protocol [26]. The previous version of the programme included three components and has been evaluated in two cluster-randomised trials [33, 34]. The fourth component introduced in the third trial was a self-test of T2D risk, the FINDRISC test [35], for parents. The HSSP was evaluated as a cluster-randomised controlled parallel trial, with randomisation at school level. Schools in mid-Sweden with a higher proportion of parents with low education than the national average were invited to participate. In the 17 schools (eight intervention, nine control) that accepted the invitation 353 families consented to participate in the HSSP intervention.

To identify the sample for this study a purposeful sampling strategy with maximum variation was used. With the aim to enhance transferability and to select information-rich cases, an effort was made to achieve maximum variation [32, 36, 37], among the following characteristics: parents’ region of birth, the sex of the parent, the child’s weight status, the sex of the child, and school of the child. Parents who were unable to express themselves in Swedish were excluded. An inclusion criterion was that either the interviewee or the co-parent had attended the MI-session. One of the eight intervention schools was excluded from the study, as the MI-sessions were not provided in that school. Two of the authors (MEM and ÅN) identified the eligible parents based on maximum variation of the selected characteristics. Parents were approached by telephone (by MEM) and gave the first consent orally. In the second step, the parents received an invitation sent by email and a written consent form was sent home. The interviews were scheduled based on suitable days and times for the parents and further material (the brochure and the workbook with home assignments) used in the intervention was sent by email as a reminder prior to the scheduled interview. In one of the schools, only few parents could express themselves in Swedish. One parent that was identified and chosen did not respond to the phone call or the invitation sent by email and therefore this school was not included in this study. Two other parents selected were abroad and could not attend the interviews. These three parents were replaced with parents with similar characteristics from the six remaining intervention schools. The family was classified as “Born outside the Nordic region” if one or both parents reported their country of birth being other countries than Sweden, Finland, Norway, Denmark or Iceland.

### Data collection

Twenty semi-structured telephone-based interviews were performed and audio-recorded, the length of the interviews varied between 18 and 60 min per parent. An interview guide (Supplementary material) was developed by MEM, ÅN and SA and included all domains in the CFIR: Intervention characteristics, Outer setting, Inner setting, Characteristics of individuals, and Process [27]. Examples of questions were: “How come you as a family chose to participate in HSSP?”, “What do you think worked well/less well?”, “How relevant were the different parts of HSSP for your family?”, “How do you think the communication between you and the school has been?”. The questions were pilot tested with a parent in the HSSP intervention group who was not included in this study, and the interviewer (MEM, female, not known to participants) used probing when appropriate and adapted the language used to fit the interviewees knowledge of Swedish. In order to decrease participant burden, the parents were not asked to review transcripts. The interviews were audio-recorded by the interviewer and transcribed verbatim by an external consultant.

### Ethics approval and consent to participate

The HSSP study was approved by the Regional Ethical Review Board in Stockholm No. 2017/711 − 31/1 and conducted in accordance with the Declaration of Helsinki. Oral and written informed consent was collected from the participating parents. The names of the participating parents were replaced by numbers to ensure anonymity in the result section.

### Data analysis

Qualitative content analysis [39] was used to analyse the data with a deductive approach as the primary analysis, conducted as described by Elo and Kyngäs [40]. The CFIR framework constituted the theory on which the deductive, theory-driven analysis was based.Thus, the constructs in each of the five domains of the CFIR were used as the categorisation matrix onto which the data were coded in the deductive analysis to identify barriers and facilitators to participating in the HSS intervention as experienced by the parents. However, in order to inform theory further, data that did not correspond to any of the CFIR constructs but carried information on parents’ perceived barriers and facilitators to participating in the HSS intervention, were marked and inductive analysis was undertaken with that data. Both the deductive and inductive analyses were kept on a manifest level. The analysis process was conducted as follows: first, in order to create a common understanding of the CFIR constructs as a categorization matrix, the three analysists (MEM, ÅN, and SA) thoroughly and independently read the CFIR description of domains and constructs, and then discussed to apply the constructs to data. Second, each one of the analyst independently applied the constructs to one interview each where text were marked and meaning units in the text were identified as corresponding to one CFIR construct, and representing a barrier or facilitator to participating in the intervention. Thereafter, the analysts compared the identified meaning units and corresponding CFIR constructs and discussed the application of the constructs as categorisation matrix. Third, after a consensus understanding of the CFIR constructs as categorisation matrix and its application on data corresponding to barriers or facilitators to participating in the intervention (see Table 1 for application of CFIR constructs in the analysis), all three analysts applied the CFIR construct categorisation matrix to three interviews independently, by marking data of importance to the study and identifying meaning units corresponding to one of the CFIR constructs in each chunk of data. The analysts subsequently discussed the CFIR constructs that had been identified in the data, and data which did not correspond to any of the CFIR constructs but still carried information on barriers and facilitators to participating in the intervention. Fourth, MEM subsequently applied the categorisation matrix to the remaining interviews, where ÅN and SA peer-reviewed the process by reviewing the coding and discussing difficulties that MEM identified in data. After reaching consensus on the deductive analysis process and the CFIR constructs identified in data, the three analysists continued the analysis with the data that carried information regarding barriers and facilitators to participating in the intervention but which did not correspond to any CFIR construct. Here, an inductive analytical approach was applied using the process as described by Elo & Kyngäs [40]. Finally, all three authors discussed the inductive codes, and revised the titles of codes in consensus. To be consistent in vocabulary, “constructs” have been used throughout the descriptions of results, both regarding theoretically and empirically derived results. Microsoft Excel 365 was used and during the analysis process notes were taken, and quotes were highlighted. MEM is a PhD student with a clinical background as a dietitian with many years of experience of working with parents and children both individually and in group. Associate professor ÅN, who is an anthropologist with training within behavioural science, has extensive experience in family-centred health promotion and qualitative research. SA is an anthropologist and public health scientist with expertise in qualitative research and health promotion directed to children and families, with a focus on health-related behaviours and mental health.

To guarantee anonymity, the interviewees were assigned by their parental role (F = father, M = mother) and numbers (1-20). The explanation of the context and nonverbal communication of the citations are written in square brackets [X]. The interviews were conducted and transcribed in Swedish and after reaching consensus among authors, selected quotes were translated into English.

## Results

In total 20 parents participated in this study; their characteristics are summarised in Table 2.

**Table 2:** Descriptive characteristics of the participating parents and their children

	Total *n* = 20
**Parental demographics (n)**	
Fathers	10
Born outside the Nordic region^a^	14
High education^b^	15
Participated in MI-session^c^	18
Conducted the T2D risk-test^c^	6
**Children’s demographics**	
Boys (n)	9
Age (years), mean (SD)	6.3 (0.3)
*BMI status*^*d*^ *(n)*	
Underweight	1
Normal weight	13
Overweight	4
Obesity	2

^a^One or both parents born outside of the Nordic region

^b^Highest reported education level of either of the parents defined as high; > 12 years and low; ≤ 12 years

^c^Intervention component of HSSP

^d^Defined according to IOTF cut-offs [38]

The deductive analysis identified eight constructs which aligned with the CFIR constructs describing the parents’ experiences of barriers and facilitators related to participating in the HSSP intervention. In addition, in the inductive, empirically-driven, analysis two additional constructs were identified which are not included in CFIR; Social factors and Cooperation. The constructs with corresponding facilitators and barriers in both the deductive and the inductive analyses are presented in Table 3.

**Table 3:** Deductive constructs with corresponding barriers and facilitators, theoretically derived from CFIR, and empirically derived constructs to inform theory developement

Theoretically derived results derived from the deductive analysis based on CFIR	
*Domains* & Constructs of CFIR	Facilitators (F), Barriers (B)
*Intervention Characteristics*	
**Evidence Strength & Quality**	**F**: Trust in positive intervention outcomes, Trust in research behind the intervention **B**: Skepticism towards intervention component
**Adaptability**	**F**: Tailoring to suit specific needs **B**: Lack of tailoring to suit specific needs, Not sufficient time to finish
**Complexity**	**F**: Understandable, easy, on the right level
**Design Quality & Packaging**	**F**: Right focus, Exciting, Relevant, Good information, Presentation of the material **B**: To comprehensive, Missing out due to lack of good packaging, Only printed format
***Outer Setting***	
**Needs & Resources of Those Served by the Organization**	**F**: Health talks, Being kept updated, Source of inspiration, Pre-knowledge **B**: Not being susceptible, Having enough knowledge
***Inner setting***	
**Available Resources**	**F**: Commitment/Involvement, Trust in the good-will of the school **B**: Lack of commitment/Involvement, Lack of effort
***Characteristics of Individuals***	
**Knowledge & Beliefs about the Innovation**	**F**: Acquired knowledge, Reminders regarding healthy behaviour, Confirmation **B**: Falling back into old habits
***Process***	
**Executing**	**F**: Whole family involved, Health information shared with others **B**: Limited possibility to engage, Missed out
**Empirically derived results to inform theory development of CFIR**	
***Empirically derived constructs***	
**Social factors**	**F**: Cultural adaptation and integration **B**: Bad economy, Does not suit parent’s everyday life, Societal transition, Lack of integration
**Cooperation**	**F**: Functioning relationship and cooperation, Functional and sufficient information-flow, Division of responsibility, Arouse of interest in school **B**: Lack of cooperation, Lack of communication, Communication through 6 year-old, Dysfunctional relationship

### Theoretically derived results from the deductive analysis

#### Domain: Intervention characteristics

This domain contained parents’ descriptions of the characteristics of the components of the HSSP intervention (the brochure, the class-room activities, the workbook, the MI-session with the school-nurse and the diabetes risk test). The findings related to the importance of adapting of the intervention to fit the abilities of the parents, to meet needs and enhance trust and understanding.

##### Construct: Evidence strength & quality

Parents described their confidence in the quality and legitimacy of the intervention. Facilitators related to the parents’ trust in the research on which the intervention is based, and that the intervention would have positive influences on their children’s the health, e.g., parents described how the scientific background of the intervention changed their attitude towards participating:

*“To be honest…At the first meeting… At first when you started talking about this, I thought, “This is not for me”, but when I was listening to what this is all about and that it’s about the children and that… you will be helping the children to be healthy, then it was interesting to me.“ (M20)*.

However, other parents also expressed a lack of trust e.g., that they did not trust the result of the diabetes risk test, which indicated that they had an elevated risk to develop T2D:

*“Yes, we have both done it and then the result came that we both have a risk of developing it or that we have the risk of becoming diabetic. Yes, but I don’t trust it.” (F2)*.

##### Construct: Adaptability

Facilitators identified were the adjustments or tailoring of the different parts of the intervention that made it more accessible and enabled the parents’ engagement, e.g., that the school-nurse was flexible when booking the time for the MI-session or that they could receive the brochure in their native language.

*”I was the one who got to direct the conversation [the MI-session]. It wasn’t like she was in charge of it, but I kind of got to talk about what I was experiencing and if there was anything that we could change and improve.” (M4)*.

Barriers included a lack of flexibility or tailoring to fit family needs. Parents expressed that they did not have the opportunity to attend information meetings or had insufficient time to finish the workbook assignments.

*“What was a little difficult was that when you kind of had to show for a week like how much screen time you have and how you’ve gotten to and from school. And then you kind of get the book [workbook] home on Thursday or Friday, and it has to be returned on Wednesday the week after and then it feels like it kind of doesn’t … you kind of didn’t have a whole week to do that [the assignments].” (M13)*.

##### Construct: Complexity

In relation to this construct, only facilitators were identified. The parents expressed that the content in the materials used was easy to understand and that the assignments were easy to complete. In general, the parents perceived the degree of complexity to be on the right level:

“For me, it was easy to understand.” (F2).

##### Construct: Design quality and packaging

Facilitators in this construct were related to the appreciation of the different parts of the intervention. Parents commented on parts they themself or their family members thought were good, e.g., that the workbook with assignments was fun and exciting, that the diabetes test was relevant and added value and that the brochure contained relevant and good information. They also described that they appreciated the MI-session and how the school-nurse treated them. Additionally, the parents expressed their thoughts about the presentation of the different parts e.g., that they appreciated getting the brochure and the home assignment as a paper copy, although this was also mentioned as a barrier. Parents mentioned that they would have liked a digital format as well, because the material was lost or thrown away. A parent who preferred the printed version expressed:

*”I’m an old-fashioned person in that way […] I like printed things because then you can go back and you have that. I read it more carefully when I have it printed.” (F7)*.

Further barriers related to the intervention being too comprehensive and that some parts could have been packaged better e.g., the diabetes risk test, which passed unnoticed as they weren’t reminded to fill in the test, or MI sessions that were too long:

*”The talk was about 40 minutes to 1 hour and I have things that I didn’t even write down so maybe limit it to half an hour because […] I feel that it’s enough.” (M20)*.

### Domain: Outer setting

In this domain the construct ‘Needs & resources of those served by the organization’ was touched upon. The findings related to the need of follow-ups, reminders and to get relevant information connected to health matters continuously.

#### Construct: Needs & resources of those served by the organization

It was perceived to be beneficial to have talks about the child’s activity and dietary behaviours as the child gets older in order to alter or promote their child’s healthy behaviour. Parents expressed a wish to have continuous health talks with the school nurse or to get the opportunity to talk with other parents to get tips, information, and advice. They mentioned that they would like to be updated about things related to their child’s health:

*”Like, Go! ‘Now they have opened a great new outdoor gym near you or near the school, go there and try’. So that you can […] as a parent get tips on maybe thinking differently. Yes, on one hand there is lack of time, then you are quite comfortable and want the information to come to you.” (M13)*.

Parents also described facilitators of pre-knowledge about health matters, e.g., that they had learnt about healthy behaviours in other contexts, and that they had diabetes or other illnesses within the family and could therefore relate and were well-equipped. This was also described as a barrier, other parents expressed themselves not needing the information given as they already felt that they had everything under control and had enough knowledge in health matters:

*“I don’t think we’re in a great need for such conversation. Most of what was said in the conversation we had already read or learnt.” (M6)*.

### Domain: Inner setting

This domain consisted of matters related to the school-setting in relation to carrying out HSSP and only consisted of one construct: Available resources. The findings related to the importance of the commitment, effort and the appearance of the intended good will of the school staff who delivered of the intervention.

#### Construct: Available resources

Parents described facilitators in the form of the commitment of the school, the school staff and the resources they provided during the intervention. They also described their trust in the school’s will of wanting their children to be healthy e.g., by providing healthy food in school and engaging their children in physical activity during school hours. A parent described:

*“I don’t know much about it, but I know they’ve kept track of it [the intervention] every week anyway. They seem to take it seriously, working on it and things like that. [The child] has talked about that they’ve been working on it…” (M6)*.

Barriers mentioned by parents were the lack of commitment and effort from the school, and the school staff not providing a kick-off meeting or opportunities for the parents to get more engaged in their work with the intervention. Parents described that the school-nurse was the one most involved, and that some teachers were more committed than others:

*“So when it comes to the teachers, they have been completely uninterested and haven’t shown much commitment to this [the intervention], so to speak. […] It is the nurses […] they are the ones who have been primarily involved in this and…the head teacher…” (F9)*.

### Domain: Characteristics of individuals

This domain contained the parents’ description of matters in relation to the intervention from their own point of view, and only included one construct. The findings related to the importance of the repetition of health information to confirm existing knowledge and to remind parents about healthy behaviours.

#### Construct: Knowledge & beliefs about the innovation

Parents described facilitators in relation to their positive attitude and thoughts about the intervention. They believed that they had acquired knowledge or that they had received confirmation and reminders regarding healthy behaviours. Parents also mentioned that children should be able to take part of the intervention at a young age. A parent expressed contentment:

*“I knew everything before and stuff, but I’m glad you care and do this for the sake of the children.” (F3)*.

One obstacle mentioned by parents was that although there was a focus on healthy behaviour during the intervention, it was easy to fall back into old habits:

*“This information booklet.it was relevant, but unfortunately you read it and it lasts for a maximum of two to three weeks and then you kind of fall back a little”. (F13)*

### Domain: Process

This domain consisted of the parents’ description related to carrying out the intervention and comprised of the construct ‘Executing’ as the data only included statements related to this construct. The findings relate to the importance of encouraging and facilitating the involvement of the whole family and key people around the family.

#### Construct: Executing

Parents reported facilitators in relation to how the intervention was carried out by the child, the parents, and the whole family. They expressed how they executed the intervention tasks together, how they read and discussed health matters connected to the intervention and how they shared the information with other people in their surroundings, such as family members, friends, and neighbours:

*“I have read this and then I have told the father and we talked at home too […] And then my eldest son read it, and I told him that “you can read it too and if I do something wrong you can tell me”. So he read it and it was great, yes…we talked about thinking about the food too […] why they eat this food.” (M14)*.

A barrier expressed was that only one parent had the opportunity to engage in parts of the intervention, e.g., the MI-session. Parents also mentioned that they hadn’t taken part in the intervention very much and that they e.g., hadn’t seen the brochure or the workbook at all. A parent expressed missing out:

*” She’s the one who read it. I was going to read it, but I didn’t get the chance.” (F17)*.

#### Empirically derived results from the inductive analysis – to inform theory development of CFIR

These constructs were found in the data but could not be directly encoded in CFIRS’ existing structure of domains and constructs.

### Construct: Social factors

It includes prerequisites that influence the parents’ participation in the intervention and the parents’ perceptions of socio-demographic circumstances. Parents described how different social conditions were important and played a role in matters related to health e.g., migration, the area one lives in, cultural background, food culture and economy. The findings could explain unexpected positive impacts of the intervention, but also that circumstances related to the wide variation in social and cultural conditions can complicate the execution of the intervention as planned.

Aspects that the parents described as facilitators were other needs being fulfilled through the intervention, e.g., integration, where parents described that even though they could have received the brochure in their native language they preferred to receive it in Swedish as they expressed that they wanted to learn Swedish:

*”No, no, it was very simple. For those of us who don’t know Swedish, it was also easy to read. It is absolutely important; I can’t read so much text […] This type of text works well.” (F12)*.

Further, parents described that they appreciated their current societal conditions. One parent expressed how important it was that her children knew that they were living in a privileged society:

*“I want them to know more… that when they eat food, that they take as much as they can eat… Because they don’t understand that they’re having it great here. There are many children they go to bed without food…Sometimes when we sit and tell them things like…"yes there are people who don’t have food, you should be grateful that you have food, a home, and a family.you have everything. You have the right to go to school and there is everything you need.“ (M14)*.

A barrier that parents expressed was having financial constraints, which was described as an obstacle for signing up the children for different activities:

*”We don’t have it easy financially now…she likes activities…she is very interested in gymnastics, and she likes to swim…she likes to dance. Yes, but we can’t give her much opportunity […] I want to enrol her in the gymnastic courses, but when I think about the money.no, I just explain to her, well she couldn’t attend the course.” (M1)*.

Another barrier that parents described was that some of the assignments did not fit their everyday life, e.g., that it was burdensome to fill in the workbook every day and that they had a heavy workload and could not prioritise the assignments. Other barriers were related to the transition they have gone through by migrating to Sweden, e.g., not knowing Swedish well enough, having a different food culture and also how they were used to having more contact with the teachers in their home country than in Sweden. A parent expressed difficulty as his child preferred Swedish food which they don’t eat at home:

*”Some vegetables [The child] does not want to eat vegetables at home, she just wants fruit. But at school it works well, but sometimes at home she says “I don’t want to eat this food” or “I want something else, like Swedish food”. We cook at home only Persian food, we can’t cook Swedish food.” (F2)*.

### Construct: Cooperation

Cooperation describes the interaction between the school and the parents, and the parent’s view of their responsibility to reach positive outcomes.

The parents described a functioning interaction and cooperation between themselves and the school staff as a facilitator, of change for the children and their health. They expressed a well-functioning flow of information as a facilitator, further that the communication mostly went through the child, and that this was sufficient and effective. Parents also described that they perceived the cooperation with the school as a facilitating factor since their children spend a lot of time in school and are influenced by the school environment, e.g., what they eat, and activities they perform. Parents expressed their views on the distribution of responsibilities:

*“I think it’s like 50/50 that both parties should do something. We as parents cannot carry out this whole process unless the school does its part […] So I think there’s a very big responsibility on us as parents and on the school so that they implement this the way they should.” (M20)*.

The communication between the school staff and the parents also acted as a barrier. Parents described that they did not perceive the cooperation with the school as sufficient or that the school did not communicate well enough and did not take the responsibility they ought to e.g., that they wanted to know more about what they had done in school during the intervention period. Further, the information-flow through the child did not always function well, causing it to be a barrier, and that parents thought that a 6-year-old cannot take that responsibility.

## Discussion

The aim of this study was to describe parents’ experiences of barriers and facilitators related to taking part in the HSSP programme, a parental support programme carried out in the school setting in preschool-class. The findings of the deductive analyses using CFIR revealed that parental experiences of barriers and facilitators to participating in the HSSP were identified in two new constructs not included in the current version of CFIR: Social factors and Cooperation. Taken together, the findings pertaining to parental experiences highlighted several aspects of importance when conducting parental support programmes in the school-setting. These findings were related to the importance of: (1) Adaptation of the intervention to parents abilities with different social and cultural backgrounds; (2) follow-ups, reminders and regular provision of information connected to health matters; (3) the commitment and efforts of the deliverers of the intervention; (4) the need for repetition of health information given by the deliverers of the intervention; (5) encouraging the whole family and key people around them to get involved through the intervention activities; (6) being aware that the intervention may have unexpected impacts and that circumstances related to wide variation in social and cultural conditions can complicate the execution of the intervention; and (7) the cooperation and a well-functioning interaction between parents and deliverers of the intervention. These findings may help to improve implementation and fidelity to the HSSP components and other similar school-based parental support programmes.

### Different ways of obtaining parental involvement

Involvement of parents and the whole family is key in interventions aiming to promote healthy behaviours among young children. Despite all efforts, involving parents and families is challenging [25]. The involvement of parents can take place either directly or indirectly [41]. An example of direct involvement of parents is attending education sessions or behaviour counselling [42]. Indirect involvement takes place when parents do not get to meet the deliverers of the programme directly e.g. sending home health information or homework assignments. Moreover, it has been reported that face-to-face- or telephone counselling is more effective than group education or written information sent home when aiming to change children’s behaviour [43]. When developing the HSSP, we aimed to achieve high acceptability by both the children, their parents, and the teachers and both direct and indirect involvement of parents was imbedded in the programme [26]. In order to appeal to parents with different educational backgrounds, the material was written in easy-to-read Swedish, and the brochure was translated into different languages (e.g., Arabic and English) for parents who’s knowledge of Swedish was low. The findings of this study indicated that most parents thought that the intervention was on the right level and that it was easy to understand the information that was given, regardless of their background. Parents described that they appreciated the intervention, and that they acquired knowledge and inspiration related to healthy behaviours. Furthermore, these findings are similar to those reported from a school-based obesity prevention intervention in England, where parents and children valued the intervention and described changes in knowledge, skills and lifestyle behaviours [44]. A qualitative study with the aim of identifying possible ways of involving parents in another school-based obesity prevention intervention concluded that homework which involved experience-based activities and were fun and novel may increase awareness of healthy physical activity and diet and were enjoyed by both children and parents [45]. In line with this, one of the components that was appreciated by both children [46] and parents participating in the HSSP was the workbook with home assignments. The intention with the workbook was that the parents would get a chance to practice role modelling and positive parenting practices and to get more involved in the programme [26]. This is in line with the Social Cognitive Theory were the importance of observational learning and parental role modelling are central mediators [47]. Parents in this study also expressed a need for repetition of health information. Such repetition could be achieved through extending the intervention across the child’s schooling with e.g., yearly recurrent MI sessions with the school nurse, and additional information to parents and activities for children. This type of repetition over time could likely contribute to sustaining healthy behaviours across child development and facilitate practice of positive parenting adapted to the child’s level of development through recurrent information and motivational conversations.

### Social factors and Cooperation

Two constructs that were identified, and not included in the current version of CFIR were Social factors and Cooperation. Although the domain ‘Characteristics of individuals’ was originally directed at the deliverers of the intervention, [27], in this study parents were considered as the deliverers of the intervention to their children (Table 1). Therefore, the two factors identified could be seen as additional contructs to this domain of CFIR. Another qualitative study involving parents and teachers from previous version of the HSSP emphasised the need for increased cooperation both within the family and between school and the family and also suggested an expansion of the CFIR to accommodate more micro-organisational levels [48]. This study further strengthens the need for an expansion of the number of CFIR constructs in this domain to be able to fully capture the parents’ view of the intervention. In line with our finding,, an update of CFIR is ongoing and in line with this suggestion [49].

Other social aspects such as migration, social integration, food culture, economy, workload etc. were found to have an impact on the parents wish to participate in the intervention and should be taken into consideration since these aspects may influence their degree of involvement. One barrier that parents described was financial constraints, e.g., in relation to not being able to sign up their children for different activities. A study exploring challenges of low-income parents to participate in an obesity prevention intervention reported parents’ inability to find affordable family activities outside the home [50]. Furthermore, in another study conducted in Australia, parents commented that their children were prevented from participating in sporting club activities due to the lack of affordability of membership fees [51].

The construct Cooperation highlights the need for a functioning interaction and a division of responsibility between school and the parents to reach positive health outcomes for the children. These findings are in line with those from previous process evaluations of the HSSP where the importance of a clear communication between parents and teachers was emphasised [29] and further the cooperation between parents and the school [30]. In a study in four countries (Norway, Spain, Hungary and Belgium) parents considered the promotion of healthy eating as mainly a task of parents with support from the school, whereas they considered the promotion of physical activity as a shared responsibility between schools and parents [52]. In our study the parents did not express any difference between promotion of healthy eating or promotion of physical activity in relation to their view of shared responsibility with the school. The findings suggested that the parents believed that the shared responsibility with school was important and that they welcomed it.

### Strengths and limitations

Using the CFIR helped to shed light on important aspects throughout the data collection, analysis, and interpretation of the results and enhance the transferability of our findings. In addition, the approximately even distribution between participating mothers and fathers is a strength of this study. Moreover, using purposeful sampling with maximum variation of the specific characteristics of the parents, the transferability of the findings may be further increased. However, even though schools were recruited from socioeconomically disadvantaged areas, the sample was skewed towards higher educated parents thus limiting transferability of results to families of lower SEP [39]. During the analysis independent co-coding and discussions between MEM, ÅN and SA were conducted to strengthen the credibility [32]. The description of the data collection, and the use of the COREQ-checklist, may enhance the trustworthiness and transparency of the results [37]. Using a larger participant sample could have captured additional perspectives of the parents’ perceptions.

### Implications for practice

This study has several implications when taking similar parental support programmes to practice. To improve outcomes of such interventions further the following points are important to consider:

- Adaptation of the intervention to meet needs and enhance trust and understanding among parents with different social and cultural backgrounds.
- Enhance the potential impact of the intervention with follow ups, reminders and keeping the participants informed with relevant information.
- The commitment and effort of the deliverers of the intervention.
- The repetition of health information to confirm existing knowledge and to remind parents of healthy behaviours.
- Encourage and facilitate the involvement of the whole family and key people around the family.
- Be aware that the intervention may have unexpected impacts on integration in society.
- The cooperation and a well-functioning interaction between deliverers and receivers of the intervention.

## Conclusion

Parental experiences of barriers and facilitators towards participating in the parental support intervention highlighted that the commitment of the school staff and a well-functioning interaction between the school and the parents is crucial for participation. Moreover, involvement of both parents and other key people around the child is important. Adaptation of the intervention on the right level, follow-ups, and continuous delivery of relevant health information needs attention. Furthermore, it is important to have in mind that the intervention may have unexpected positive impacts regarding integration of migrant families on the one hand. On the other hand, circumstances related to variations in social and cultural conditions can be a barrier to the execution of the programme. The findings of this study can contribute to improve parental involvement and to better planning, execution, and implementation of family support interventions.

## Electronic supplementary material

Below is the link to the electronic supplementary material.

Supplementary Material 1

## Data Availability

The datasets generated and/or analysed during the current study are not publicly available due to the quest to protect the confidentiality of the participant but are available from the corresponding author on reasonable request.

## List of abbreviations

HSSP: A Healthy School Start Plus
SEP: Socioeconomic position
SCT: Social Cognitive Theory
T2D: Type 2 diabetes
CFIR: Consolidated Framework for Implementation Research
MI: Motivational interviewing
