# Supplementary material for: Parents’ experiences of participating in the Healthy School Start Plus programme – a qualitative study
Source: BMC Public Health. 2023 Apr 4;23:646. doi: 10.1186/s12889-023-15552-8 (PMC10074815; doi:10.1186/s12889-023-15552-8)
Supplement: Supplementary file 1 — Supplementary Material 1 [file 12889_2023_15552_MOESM1_ESM.docx]

# Interview guide

**Introduction**

- What come you as a family chose to participate in Healthy School Start Plus (HSSP)?
- How do you think the project has responded to (met) your expectations?
- How did you feel that HSSP worked in general? What worked well/less well?

**Content**

- How did you find the brochure?
- What is the difference between digital and printed material?
- How did you find the MI session?
- In what manner did you find the MI session tailored to your specific family?
- How do you feel that the school nurse supported you in reflecting (exploring your own thoughts) about your child's habits and needs around this at home?
- What could the school nurse has done differently? What would need to be adapted/changed to make it better/easier (for your family)?
- How did you find the child’s workbook?
- What did your child think of working in the workbook?
- What would you have needed to facilitate the work at home with the workbook?
- How did you find the diabetes-risk test?
- Did you do anything different based on the result you got from the diabetes-risk test?

**School:**

- How do you experience the school's involvement in the HSSP?
- How do you think the communication between you and the school has been?
- When it comes to food and movement, what responsibility do you think you as parents have in comparison to the school to ensure that the different parts of the project are implemented?
- How do you feel the information and communication about the HSSP has been?

**Overall questions:**

- How relevant were the different parts of HSSP for your family? What would have been more relevant?
- What would you need to be able to continue with the changes that have been made?
- What would you need to get even more support regarding food and movement?
